# Dynamic Pupillary Response in Multiple Sclerosis Patients with and without Optic Neuritis

**DOI:** 10.3390/biomedicines11123332

**Published:** 2023-12-17

**Authors:** Amparo Gil-Casas, David P. Piñero, Ainhoa Molina-Martín

**Affiliations:** 1 Optometric Clinic, Foundation Lluís Alcanyís, University of Valencia, 46020 Valencia, Spain; 2 Group of Optics and Visual Perception (GOPV), Department of Optics, Pharmacology and Anatomy, University of Alicante, 03690 Alicante, Spain

**Keywords:** multiple sclerosis, optic neuritis, pupillometry, dynamic pupillary response

## Abstract

Multiple sclerosis (MS) is a neurodegenerative disease that affects the central nervous system which produces abnormalities in visual function, as disturbed pupillary responses, even after an episode of optic neuritis (ON). The aim was to assess different parameters of the pupillary response in MS subjects with and without ON. Therefore, 24 eyes of healthy age-matched subjects were included, 22 eyes of subjects with MS (MS group), and 13 subjects with MS with previous ON (MSON group). Pupillary parameters (ratio pupil max/min; latency; velocity and duration; contraction and dilation; and amplitude of contraction) were recorded with the MYAH topographer. Statistical analysis was performed by IBM SPSS Statistics, and parametrical or non-parametrical tests were used according to the normality of the data. MS patients did not significantly differ from healthy patients in any of the parameters analyzed (*p* > 0.05). Only patients with previous ON were different from healthy patients in the amplitude (40.71 ± 6.73% vs. 45.22 ± 3.29%, respectively) and latency of contraction (0.35 ± 0.13 s vs. 0.26 ± 0.05 s, respectively). The time to recover 75% of the initial diameter was abnormal in 9% of the MS subjects and 12% of MSON subjects. Based on the results of this study, the contraction process, especially latency and amplitude, was found to be affected in subjects with MS and previous ON. The degree of disability and the relation of the decrease in pupil response with other indicators of MS disease should be further investigated considering other comorbidities such as ON in the affection.

## 1. Introduction

Multiple sclerosis (MS) is an unpredictable neurodegenerative disease that affects the central nervous system (CNS). It is an acquired, progressive, inflammatory, and autoimmune demyelinating disease which is characterized by irregular exacerbation, followed by total or partial remissions [1]. MS patients present a multitude of dysfunctions, among which visual impairment is one of the most common [1,2,3,4]. Lesions in the CNS occur at different times and in different locations, producing a variety of neurological symptoms among which dysfunction of the autonomic nervous system (ANS) is very common [5]. Indeed, between 45% and 84% of MS patients exhibit autonomic dysfunction [6,7]. Even in the early stages of MS, non-specific impairment of the central pathways can affect the autonomic nervous system and affect functions such as the pupillary response, sleepiness, and fatigue [8,9,10,11]. ANS is a component of the peripheral nervous system that regulates involuntary processes such as heart rate, blood pressure, respiration, and pupillary responses [12]. It mainly consists of two divisions: the sympathetic nervous system (SNS) and the parasympathetic nervous system (PNS). If the focus is on pupillary function, pupil size is controlled by these two pathways that, although interconnected, are considered distinct: the parasympathetic constriction pathway, which innerves the iris sphincter muscle, and the sympathetic dilation pathway, which innerves the iris dilator muscle.

The constriction pathway begins when the light reaches the retina, where the intrinsically photosensitive retinal ganglion cells (ipRGCs) that are responsible for pupillary activity are located [13,14]. Through the optic nerve and after crossing the chiasm, information arrives at the pretectal nucleus (PN). From the PN, information is sent to the Edinger–Westphal nucleus (EWN), then the information is sent via the Oculomotor Nerve (III) to the ciliary ganglion (CG) to stimulate the iris sphincter muscle and produce miosis. The dilation pathway is understood less than the constriction pathway. It is a subcortical pathway that starts at the hypothalamus. It projects to the intermedio-lateral column (IML) which projects to the superior cervical ganglion (SCG), located just outside the spinal cord. Then, the SCG projects, via a complicated network of nerves, to the iris dilator muscle [15].

Pupillary disorders, such as relative afferent pupillary defects, are present in around 20% of MS cases [16]. Other disorders, such as Marcus Gunn [17,18], Argyll Robertson with lesions in the area of the Edinger–Westphal nucleus [19], and cranial nerve III dysfunction, were also described in the 1990s [20,21,22]. Although abnormalities have been found in the pupilar light reflex (both constriction and dilation), impairments in the parasympathetic system are more prevalent than in the sympathetic pathway [23,24]. Hence, a reduction in the constriction amplitude and an increase in latency have been most frequently reported [24]. Velocity, although less predominant, can be reduced, especially during contraction [25,26]. When lesions of the optic nerve occur, abnormalities in the pupillary response may appear in the symptomatic eye [27]. Even if the visual acuity improves almost completely in MS patients with optic neuritis (ON) after the resolution of the neuritis process, reduction in pupillary sensitivity may be observed [28,29].

The pupilar light reflex (PLR) has growing interest as a biomarker of a variety of neurologic alterations, such as Alzheimer’s disease [30,31] or autism spectrum disorder [32]. The measurement of dynamic aspects of PLR can provide valuable data concerning the function of the sympathetic and parasympathetic pathways. In fact, previous studies have been carried out with different devices that allow for the quantification of the pupillary reaction to light, such as MRI [23], VEP [24,28], or specific devices [33] developed just to quantify pupillary function. These integrate an algorithm called the Neurological Pupil index (NPi) to evaluate whether the pupillary response is normal or if there is suspicion of neurological problems or traumatic brain injury [34]. These techniques allow for a correct characterization of the pupil response but require some specific instruments which are not available for most visual specialists. Additionally, functional imaging techniques such as MRI or VEP are very invasive techniques for patients.

The latest corneal topographers, used for corneal morphology analysis, have incorporated the pupillometry measures in their software based on imaging analysis. These modules seemed to provide information about pupil parameters in the same manner as pupilometers, with the advantage of combining both techniques in the same instrument. Some of these pupilometer modules have been studied in the past, validating this tool for dynamic pupil measurements, but differences in instruments (topographers) and specifically differences in the light intensity of the devices or measurement conditions made the comparison between studies difficult. Additionally, these devices have been used in normal subjects, but there are no previous studies which analyze the dynamic pupillometry measures in subjects with MS. The aim of the present study was to assess different parameters of the pupillary response in MS subjects with and without previous ON using a pupillometry module integrated into a topographer device and to compare these results with those obtained in an aged-matched sample of healthy subjects.

## 2. Methods

### 2.1. Subjects

Patients were recruited from local MS associations and were evaluated at the Optometric Clinic of the Lluís Alcanyís Foundation of the University of Valencia and the Optometric Clinic of the University of Alicante, Spain. All participants provided informed and written consent prior to the beginning of study procedures according to the Declaration of Helsinki. The protocol was approved by the ethics committees of the University of Valencia (H1527574656645) and Alicante (UA-2018-03-02).

Subjects were divided into two groups: the MS group (subjects with MS without a history of ON) and the MSON group (subjects with MS and a history of ON in the past, now resolved). Results were compared with a control group composed of the same number of healthy age-matched subjects. In the control group, patients with ocular or systemic diseases were excluded. In the MS group and MSON group, those patients with comorbidities other than MS and/or ON were excluded.

A full ophthalmic examination, including BCVA testing with an ETDRS test, intraocular pressure measurement, slit-lamp biomicroscopy, subjective refraction, horizontal visible iris diameter (HVID), and pupillometry, was performed for all of the study participants.

### 2.2. Pupilometer

**Figure 1:**
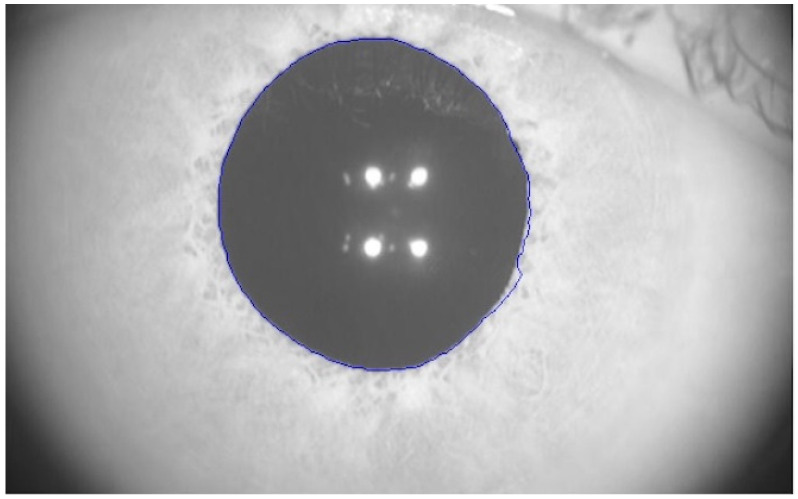
Real image taken by MYAH topographer with the pupillometry module. The pupillary diameter is automatically detected and outlined in blue.

Pupillary parameters were recorded with a module integrated into the MYAH topographer (Topcon EU, Tokyo, Japan). This module analyzes dynamic pupillometry with controlled light conditions using a central fixation LED and 2 white light LEDs for the photopic phase (1100 mcd). Pupillometric variation is monitored by four infra-red LEDs (940 nm). The total test time was 16 s: 2.5 s under low-medium lighting conditions (mesopic), 2.5 s under medium-high lighting conditions (photopic), and 11 s under low lighting conditions (scotopic). Unfortunately, the specific values for light intensity in cd/m^2^ for every illumination level were not provided by the manufacturer. Pupillary response is analyzed measuring the size of the pupil over time. This software automatically outlined the pupillary contours of the participants on the images, as exemplified in Figure 1 (blue line). The precision of the measure was ±0.05 mm.

The data provided automatically by the pupillometry module software are the maximum and minimum pupil values, as well as the pupil diameter in each fraction of time. Other parameters that allow for the characterization of the pupillary reaction to light, such as contraction and dilation latency, velocity, and duration, can be obtained from these data.

### 2.3. Parameters Measured

**Figure 2:**
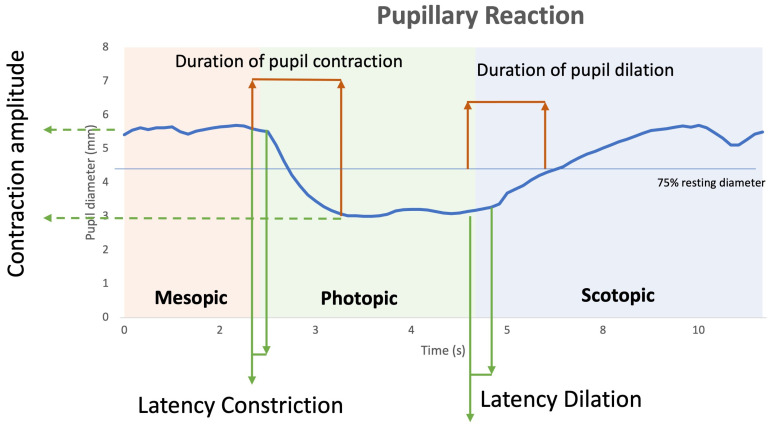
Graphical representation of the pupillary parameters analyzed. The mesopic phase is shown in orange, the photopic phase in green, and the scotopic phase in blue.

The pupilometer provides a graph similar to the one shown in Figure 2. This graph shows the variation of pupil diameter (mm) as a function of time and illumination conditions.

The parameters directly provided by the pupillometry module were the following: maximum and minimum pupil diameter, HVID, and pupillary diameter at each time fraction during the 16 s of the test. As the pupillometry module of the topographer only provides these values based on the patient’s pupillary size over time, the following parameters were calculated: (1) Ratio of pupil max/min as pupil size divided by HIVD to avoid eye size difference between subjects as indicated by other authors [28]. (2) The contraction amplitude is the pupil’s capacity for variation. It is calculated as a percentage: (Pupil Max − Pupil Min/Pupil Max) × 100. (3) The latency of pupil constriction describes the delay in pupil constriction following the onset of a light stimulus. It is given in seconds. (4) The duration of pupil constriction is the time interval between the start of the constriction and the plateau. (5) The velocity of pupil constriction, given in mm/s, is the speed at which the pupil narrows in response to light. (6) The latency of pupil dilation describes the delay in pupil dilation following the onset of a light stimulus. (7) The duration of pupil dilation is the time interval between the start of the dilation and the time it reaches 75% of the initial diameter. (8) The velocity of pupil dilation, given in mm/s, is the speed at which the pupil reaches 75% of its initial diameter after the light stimulus is turned off. A summary of the analyzed pupillary parameters and their definitions is presented in Table 1.

### 2.4. Statistical Analysis

Data were analyzed using IBM SPSS Statistics for Mac. The Shapiro–Wilk test was used to assess the normality of the distribution of variables. Hence, all the parameters examined showed a normal distribution except for contraction latency, and consequently, one-way ANOVA parametric tests were used for all variables except this one, in which the Kruskal–Wallis test was used. Although both eyes of the participants were examined, only one eye was used for statistical purposes. Due to the glare effect produced when measuring pupillometry in the first eye, the second measure could be affected. Therefore, the first eye measured was selected for analysis, that is, as per protocol, the right eye.

## 3. Results

This study included 24 eyes of healthy subjects, 22 eyes of subjects with MS, and 13 subjects with MS recovered from an episode of ON in the past. The control group included 9 males and 15 females with an average age of 49.5 ± 8.2 years and a mean corrected distance visual acuity (CDVA) of ﻿−0.08 ± 0.04 logMAR. The MS group ﻿included 8 males and 14 females with an average age of 52.9 ± 8.8 and mean CDVA of 0.00 ± 0.07 logMAR. The MSON group included 3 males and 10 females with an average age of 50.0 ± 10.3 and mean CDVA of 0.06 ± 0.11 logMAR. There were no statistically significant differences in age (*p* = 0.72), gender (*p* = 0.29), or CDVA (*p* = 0.41) among the three groups. Mean values and the standard deviation obtained for pupillary parameters of each group are shown in Table 2.

The control group showed a relative maximum pupil size slightly higher (46.38 ± 7.62%) than the MS (42.39 ± 8.32%) and MSON group (41.82 ± 8.24%), but they were still very similar (*p* > 0.05). The values relative to the minimum diameter were also lower for the MS groups, with and without ON (24.12 ± 4.50 and 23.47 ± 5.53%, respectively), compared to the control group (25.38 ± 4.34%), but did not show statistically significant differences. The time taken to reach maximum miosis was similar in the control group (1.78 ± 0.56 s) as well as in the MS (1.76 ± 0.58 s) and MSON groups (1.60 ± 0.51 s).

**Figure 3:**
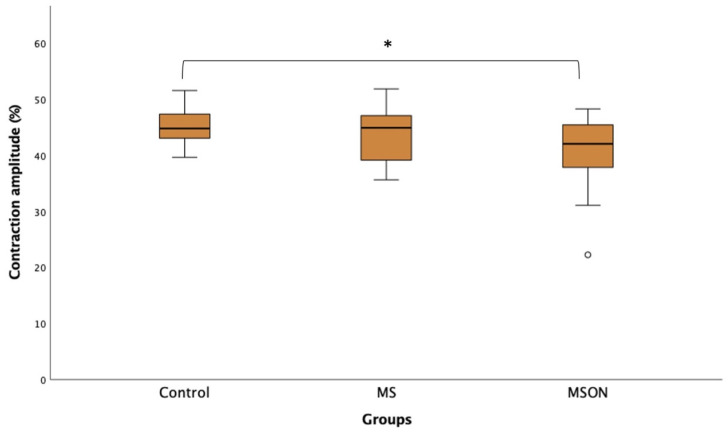
Box plot showing contraction amplitude of the three study groups. [*] exhibits statistically significant differences between control and MSON group.

The amplitude of pupil contraction in the MSON group (40.71 ± 6.73%) was reduced with respect to the control group (45.22 ± 3.29%) (*p* = 0.01) but not compared to the MS group (43.75 ± 5.02%) (*p* = 0.23). Although the control group’s contraction amplitude was higher than that in the MS group, differences between groups did not reach statistical significance (*p* = 0.70), as illustrated in Figure 3.

**Figure 4:**
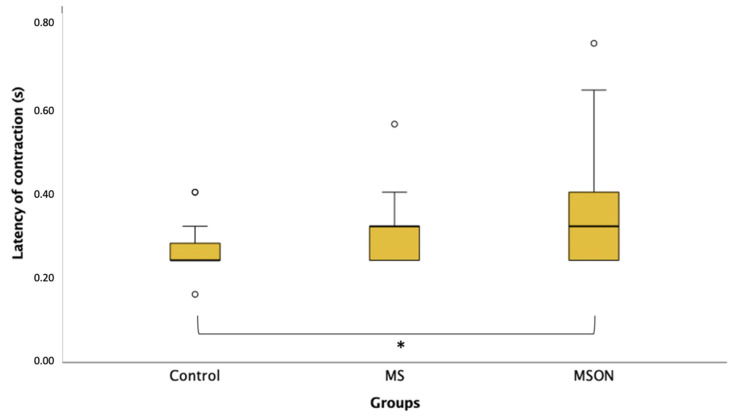
Box plot showing contraction latency of the three study groups. [*] exhibits statistically significant differences between control and MSON groups.

When comparing the latency of pupil contraction between groups, the MSON group spent more time (0.35 ± 0.13 s) than the control group (0.26 ± 0.05 s) initiating the constriction reaction (*p* = 0.03), as also illustrated in Figure 4. Although the MS group (0.30 ± 0.07 s) also evidenced a longer latency of contraction compared to the control group, these differences were not statistically significant (*p* = 0.84). The MS group and MSON group did not show significant differences in the latency of contraction (*p* = 0.10).

The group with previous optic neuritis required less time to reach maximum miosis (1.60 ± 0.51 s), but it was similar to the control group and the MS group (1.78 ± 0.56 s and 1.76 ± 0.58 s, respectively) (*p* > 0.05). The control group showed the greatest speed in the contraction process (1.48 ± 0.50 mm/s), but it was not statistically significantly more than the MS and MSON groups (1.27 ± 0.48 mm/s and 1.29 ± 0.37 mm/s, respectively) (*p* > 0.05).

In terms of the dilatation function, none of the analyzed parameters (velocity, latency, and duration) showed statistically significant differences between the three study groups. Even so, the speed of dilation was faster in the control group (0.89 ± 0.34 mm/s), with no statistically significant differences between the MS and MSON groups (0.74 ± 0.29 mm/s and 0.64 ± 0.26 mm/s, respectively). The contraction latency, that is, the time required by the pupil from the time the luminous stimulus is turned off until mydriasis begins, was greater in the control group (1.06 ± 1.00) than in the MS and MSON groups (0.80 ± 0.38 s and 0.84 ± 0.39 s, respectively) but with no statistically significant differences (*p* > 0.05).

When analyzing the duration of pupil dilatation, that is, the time to reach 75% of the initial diameter after turning off the light, it was abnormal in 9% of the MS subjects and in 12% of the MS subjects with ON. These patients presented a reduced contraction amplitude; therefore, the dilatation percentage could not reach 75% of the initial diameter. Except for these subjects, there were no significant differences between groups in the duration of pupil dilatation (*p* > 0.05). Despite this, the MSON group exhibited the shortest time to reach 75% redilation. These results could be explained by the smallest amplitude of constriction in the MSON group, which leads to a lesser redilation distance compared to the other groups.

## 4. Discussion

The pupillary response involves multiple areas of the brain and it is controlled by the autonomic nervous system [15]. Although the sympathetic and parasympathetic systems are considered different pathways, they interact together to produce the response of miosis and mydriasis. Therefore, several structures such as the Edinger–Westphal nucleus (EWN), pretectal nucleus (PN), Oculomotor Nerve (III), ciliary ganglion (CG), and superior cervical ganglion (SCG) are involved in this process. The demyelination and axonal damage that characterizes MS could cause non-specific alterations ﻿of the autonomic nervous system [6,7,10]; therefore, the pupillary reaction has been found disturbed in MS patients [23,33,35,36].

Different devices have been used by other authors to assess the pupillary reflex. In the present study, a module integrated into the MYAH topographer which provides information about the pupil diameter at each time fraction according to the illumination conditions was used. This device is a non-invasive tool, but it was not specifically designed to quantify pupillary function. It is actually a corneal topographer in which the measurement of pupillometry has been implemented. This has the advantage that the same instrument can be used for different measurements. On the other hand, because it is not specifically designed for pupillometry, there are some pupillometric parameters that are not directly provided by the instrument such as the latency, velocity, and duration of both constriction and dilation, but these parameters can be calculated from the raw data. These parameters were calculated and evaluated in MS subjects without visual impairment to assess if this response is affected due to the disease. In addition, a group of MS subjects who have suffered ON in the past were also evaluated to assess whether that inflammatory process could affect the pupillary response.

The results obtained in the present paper showed that most of the pupillary parameters in MS subjects (without ON and normal CDVA) were comparable to those of healthy age-matched controls. These results agreed with some previous studies, in which no severe pupillometry anomalies were found nor were non-specific alterations of the autonomic system highlighted [24,33]. However, other authors concluded that up to 60% of the MS patients exhibited some impairment in pupil response [23,36,37]. Others found reduced contraction amplitudes in MS patients, but in this case, subjects presented a high level of disability [37]. Because a disability test was not performed with our patients, the present results cannot be directly related to the disability. Discrepancies between studies could be due to differences in subjects’ characteristics and the degree of disability, because the disruption of the autonomic system in MS is diffuse and there is no specific pattern of related pupillary dysfunction. Perhaps the type of progression of the disease, occurrence of exacerbations (different from optic neuritis), and frequency or severity of them could also influence the results. In addition, the time since diagnosis may also affect visual system impairment and thus the pupillary response. In our sample, these factors were not taken under consideration to segregate the groups of patients, which could mask if there really is pupillary involvement in patients with MS without previous ON.

The latency, amplitude, and velocity of pupil contraction are indicators of parasympathetic activity [15] that could be disrupted by an inflammatory process in the optic nerve. Indeed, the results of the present paper showed that patients with previous ON were not comparable to healthy subjects in those functions related to the parasympathetic system. Other authors also found an affection on the pupil response of subjects with MS and previous ON [28]. Specifically, contraction amplitude was lower in the MSON group, as they also exhibited an increased contraction latency compared to healthy controls. These differences were not present when comparing the MS group without ON with the control group. These results suggest that ON in subjects with MS could cause a higher pupillary response affection than that produced by the disease by itself. According to this, other authors found alterations in the pupil response of subjects with MS with and without previous ON, and they also found a higher affection in the group with previous ON [27,28]. Others even compared the response between eyes affected by ON and those non-affected in subjects with MS and found higher affection in the symptomatic eye [27]. Paradoxically, the group that had a previous optic neuritis episode took less time to reach the minimum diameter than the control group. This is because the initial diameter of the control group was slightly larger and the amplitude was larger; therefore, healthy patients used more time because they had to decrease the pupil diameter further. Regarding contraction velocity, it was reduced in both MS groups, and these differences were not statistically significant according to our results, but other previous studies also observed delays in contraction velocity [24,25,26].

Pupillary responses innervated by the sympathetic system such as dilation latency and velocity were not very different from healthy subjects either in the MS group or with previous ON according to the results of the present paper. Even patients with multiple sclerosis showed better values in the dilation functions. The latency was shorter, although not significant, and the time to reach 75% of the initial diameter was also shorter. However, only about 10% of subjects with MS did not reach a dilation of 75% of the initial pupillary diameter. This could be because the amplitude of contraction is smaller in patients with neuritis, so if the contraction is small, then they cannot dilate to 75%; therefore, this indicates that the measurement technique is not appropriate to evaluate the dilatation response, or in this case, a delayed dilatation response in some subjects, so the results of dilatation pupil response analysis should be considered with caution. Despite this, these results could suggest that there may be some type of disturbance that produces the hyperaction of the sympathetic tone in a non-specific way, as other authors have noted [23,24,33]. The initial pupil diameter, mainly related to sympathetic innervation, was similar among groups, ﻿regardless of ON history. Previous studies identified abnormalities in the initial diameter in MS patients, but the sample presented a high neurological disability [33].

Our results are in accordance with other authors that found pupillary pathological responses in MS patients due to decreased parasympathetic tone associated with increased sympathetic tone [23,24,33]. The results of the present work revealed some pupil disturbances innervated by the parasympathetic system in patients with previous optic neuritis without visual impairment. In contrast, these disturbances were not found in patients without ON.

Although some findings are relevant and show alterations in MS patients with ON, more exhaustive studies should be conducted to assess if pupillary function could be a reliable biomarker for these patients. Regarding instrumentation, the present device, that is, a pupilometer integrated in a topographer, provides useful information about dynamic pupillometry but studies validating this technique are still scarce even in normal subjects. The lack of studies analyzing the pupillometry in MS subjects was one of the main motivations to develop the present study, but the absence of other studies to compare with our results is actually a limitation. Issues such as the degree of disability, the duration of the disease, or even retinal and optic nerve alteration could affect pupillometry. Therefore, the present study showed some limitations as pupillometric values have not been correlated with retinal nerve fiber layer thickness, the ganglion cell complex, the function of ipRGC, which is partially responsible for pupillary activity [13,14], or with colorimetric or campimetry abnormalities that could also affect the pupil response, as observed in previous studies [38]. The lack of a group with ON but without MS is another limitation, because it will allow for a better understanding of pupillary defects and for a determination of if those pupillary defects were present from the proper ON or were increased if the subject additionally suffered from MS. In the future, it should be studied whether these disturbances can occur in MS patients with and without ON but with a visual impairment due to the progression of the disease, because in the present study, all subjects maintained a good level of VA without differences between groups.

## 5. Conclusions

Based on the results of this study, the contraction process, especially latency and amplitude, were found to be affected in subjects with MS and previous ON. Subjects with MS, even with a low degree of disability and a preserved VA, showed a general reduction in pupillary response, but these differences were only significant when analyzing the results of subjects with a previous history of ON. The degree of disability and the relation of the decrease in pupil response with other indicators of MS disease should be further investigated considering other comorbidities such as ON in the affection.

## Figures and Tables

**Table 1:** Pupillary parameters analyzed and their definition.

Variable	Unit of Measure	Definition
Ratio pupil max/min	%	Minimum and maximum pupil size. To avoid eye size differences between subjects, pupil size divided by HIVD was used, as previously indicated by the authors of [28]
Contraction amplitude	%	The contraction percentage is defined as (Pupil Max − Pupil Min)/Pupil Max) × 100
Latency of pupil constriction	seconds	The time difference between the initiation of light and the onset of pupillary contraction
Duration of pupil contraction	seconds	Time the pupil takes to reach minimum diameter
Velocity of pupil contraction	mm/s	The peak value of the velocity during contraction
Latency of pupil dilation	seconds	The time difference between when the light is turned off and the onset of pupillary dilation
Duration of pupil dilation	seconds	Time to reach 75% redilation
Velocity of pupil dilation	mm/s	Velocity which reaches 75% redilation (after the constriction)

**Table 2:** Pupillary parameters (mean ± SD) obtained for each group. [*] indicates significant differences, *p* < 0.05.

	Control Group	MS Group	MSON Group	P_Control vs. MS_ P_Control vs. MSON_ P_MS vs. MSON_
Ratio pupil max (%)	46.38 ± 7.62	42.39 ± 8.32	41.82 ± 8.24	0.18 0.09 0.79
Ratio pupil min (%)	25.38 ± 4.34	24.12 ± 4.50	23.47 ± 5.53	0.91 0.29 0.48
Contraction amplitude (%)	45.22 ± 3.29	43.75 ± 5.02	40.71 ± 6.73	0.70 0.01 * 0.23
Latency of contraction (s)	0.26 ± 0.05	0.30 ± 0.07	0.35 ± 0.13	0.84 0.03 * 0.10
﻿ Duration of pupil contraction (s)	1.78 ± 0.56	1.76 ± 0.58	1.60 ± 0.51	0.84 0.41 0.70
Velocity of contraction (mm/s)	1.48 ± 0.50	1.27 ± 0.48	1.29 ± 0.37	0.23 0.29 0.98
Latency of pupil dilation (s)	1.06 ± 1.00	0.80 ± 0.38	0.84 ± 0.39	0.91 0.73 0.91
﻿ Duration of pupil dilation (s)	0.97 ± 0.47	0.85 ± 0.38	0.86 ± 0.44	0.64 0.45 0.90
Velocity of pupil dilation (mm/s)	0.89 ± 0.34	0.74 ± 0.29	0.64 ± 0.26	0.24 0.09 0.72

## Data Availability

The data collected in this study are managed by the Optometric Clinic —FLA—University of Valencia.
